# Towards a common European ethical and legal framework for conducting clinical research: the GATEKEEPER experience

**DOI:** 10.1038/s41746-024-01092-x

**Published:** 2024-04-13

**Authors:** Alessia Maccaro, Vasiliki Tsiompanidou, Davide Piaggio, Alba M. Gallego Montejo, Gloria Cea Sánchez, Jordi de Batlle, Adrian Quesada Rodriguez, Giuseppe Fico, Leandro Pecchia

**Affiliations:** 1 Applied Biomedical Signal Processing Intelligent eHealth Lab, University of Warwick, CV47AL Coventry, UK; 2 UDG Alliance (UDGA), 15 rue Fort Barreau, 1201 Geneva, Switzerland; 3 Life Supporting Technologies-LifeSTech, Universidad Politécnica de Madrid, 28040 Madrid, Spain; 4 Group of Translational Research in Respiratory Medicine, IRBLleida, Hospital Universitari Arnau de Vilanova and Santa Maria, Centro de Investigación Biomédica en Red de Enfermedades Respiratorias (CIBERES), Lleida, Spain; 5 European Alliance of Medical and Biological Engineering and Science (EAMBES), Rome, Italy; 6 University Campus Bio-Medico, Via Alvaro del Portillo 21, 00128 Rome, Italy; 7 Fondazione Policlinico Universitario Campus Bio-Medico, Via Alvaro del Portillo, 200, 00128 Roma, Italy

**Keywords:** Health policy, Outcomes research

## Abstract

This paper examines the ethical and legal challenges encountered during the GATEKEEPER Project and how these challenges informed the development of a comprehensive framework for future Large-Scale Pilot (LSP) projects. GATEKEEPER is a LSP Project with 48 partners conducting 30 implementation studies across Europe with 50,000 target participants grouped into 9 Reference Use Cases. The project underscored the complexity of obtaining ethical approval across various jurisdictions with divergent regulations and procedures. Through a detailed analysis of the issues faced and the strategies employed to navigate these challenges, this study proposes an ethical and legal framework. This framework, derived from a comparative analysis of ethical application forms and regulations, aims to streamline the ethical approval process for future LSP research projects. By addressing the hurdles encountered in GATEKEEPER, the proposed framework offers a roadmap for more efficient and effective project management, ensuring smoother implementation of similar projects in the future.

## Introduction

Digital health is revolutionizing the approach to healthcare, particularly in the context of healthy aging^1^. Through innovative technologies such as wearable devices, mobile apps, and telemedicine platforms, digital health empowers individuals to monitor and manage their health proactively^2^. This means easier access to medical consultations, real-time health monitoring, and personalised fitness programs, enabling elderly people to maintain their well-being and independence^3^. Furthermore, digital health tools facilitate the seamless sharing of health data between patients and healthcare providers, fostering more personalised and efficient healthcare solutions tailored to the specific needs of aging populations^4^. As a result, healthy aging is no longer just about adding years to life but ensuring those added years are lived in good health and vitality, thanks to the transformative potential of digital health technologies. However, the efficacy, cost-effectiveness, and scalability of eHealth interventions must be tested before large-scale adoption. In this framework, large-scale pilots (LSPs) are key as a last step in the implementation strategy towards real-world adoption of such technologies.

In this remit, the European Horizon 2020 project GATEKEEPER (GK) (https://www.gatekeeper-project.eu/) aims to connect healthcare providers, businesses, entrepreneurs, older citizens, and the communities in which they live, to create an open and trust-based arena to combine user ideas, technologies, needs, and processes, to ensure a healthier independent life for aging populations^5^. As a result, GATEKEEPER has created an open-source, European, standard-based, interoperable, and secure framework available to all developers, for the creation of combined digital solutions for personalised early detection and interventions that cover the entire care cycle for elderly citizens. The system envisaged by GATEKEEPER is broader in scope than other eHealth solutions: instead of focusing on one disease or condition, it tries to meet the heterogeneous health needs of the elderly. In this context, and with the aim of validating the project’s technical developments in real scenarios, GATEKEEPER is an LSP project organised into 8 Pilot sites in Europe (Aragon, Basque Country, Cyprus, Greece, Poland, Puglia, Saxony, and the UK [Milton Keynes and Bangor]). Up to 30 implementation trials tackling 9 reference use cases (RUCs) are being conducted (see Table 1). However, the added value of GATEKEEPER lies not only in its many implementation trials but in their pooling via a federated approach, aiming at demonstrating the effectiveness and the cost-effectiveness of Key Enabling Technologies (KETs) such as AI, big data, and Internet of Things (IoT) for the prevention of adverse events and the management of health in later life. In order to get the gears going, looking into obtaining several ethical approvals was essential (see Table 1).

**Table 1:** GATEKEEPER Ethical approvals

	Aragon	Basque country	Cyprus	Greece	UK	Poland	Puglia	Saxony
RUC1: Lifestyle-Related Early Detection and Interventions	x	x		A. Attica B. Central Greece		x	x	x
RUC2: Chronic Obstructive Pulmonary Disease Exacerbations Management	x						x	
RUC3: Diabetes Mellitus, Predictive Modelling of Glycaemic Status		x		x			x	
RUC4: Parkinson Disease Treatment Decision Support System		x						
RUC5: Predicting Readmissions and Decompensations in Heart Failure	x						x	
RUC6: Primary and Secondary Stroke Prevention		x						
RUC7: Multi-chronic Older Patient Management Including Poly-medication	x	x	x		A. Milton Keynes B. Bangor	x	x	x
RUC8: eHealth Solutions for the Management of High Blood Pressure							x	
RUC9: eHealth Solutions for the Management of COVID-19.	A. Home B. Centre			x	x			
Total ethical approvals required: 24	4	6	1	4	3	2	2	2

In fact, a project such as GATEKEEPER, which includes the use of KETs for people and, in particular, on patients suffering from pathologies of different complexity and the use of their data, requires attention to a multitude of ethical questions. The ever-increasing use of Internet of Things (IoT) and Artificial Intelligence (AI) in healthcare itself is deeply intertwined with numerous ethical challenges concerning the interrelations between “things”/machines and humans. Specifically, the use of ICT (including IoT) in applications for personal assistance presents several challenges^6^. These include the complexity and pervasiveness of the technology that users find difficult to understand, significant privacy and confidentiality concerns, difficulties in ensuring the security of personal data, the absence of a trusted framework for data protection, and a lack of transparency in data collection and processing^7^.

As can be seen from above, and as confirmed by a literature review on the subject^8^, a frequent theme in the debate on ethics, AI, and IoT, relates to privacy, and, more prominently, to the issues regarding personal data sharing and protection^9^. It has been argued that one key feature of the use of digital devices is the passive and continuous collection of information^10^, which makes it difficult for the users to feel in complete control of the sharing and use of their data^11^.

Ethical aspects of interrelations between humans and technology are even more relevant when it comes to the application of AI and IoT in the field of health and the medical sector. Health-related data necessarily touch upon the user’s identity and the most intimate sphere of their private life. Due to the sensitivity of the data^12^ and the potential consequences for the users, human control over algorithms and decision-making systems is paramount for these applications. This is further highlighted in the European Health Data Space Regulation^13^, the ambitious initiative held by the European Union aimed at improving the healthcare sector by facilitating the secure and efficient exchange of health data across member states.

Currently, although a unified legal ethical framework of reference for AI applications is still missing, there are many different regulatory efforts to address these ethical aspects, such as The Ethics Guidelines for Trustworthy Artificial Intelligence, published by the High-level Expert Group on AI, in April 2019^14^. Another important document in this field is the EU AI Act, which is expected to reach its final adoption by the end of 2024.

To safely navigate this “uncharted” territory, since its beginning, the GATEKEEPER project has established some overarching ethical principles. The list of guiding principles was informed by the principalist approach to medical ethics^15^ and the Organization for Economic Cooperation and Development (OECD)’s Privacy Framework^16^. These principles are listed below, with a comment on their relation to the four principles embedded in the Ethics Guidelines for Trustworthy AI, namely (1) Respect for human autonomy, (2) Prevention of harm, (3) Fairness, and (4) Explicability:

A. Collect the minimum required data and ensure that data processing protocols are transparent and accountable (principles 2 and 4);
B. Support the ethical capabilities of human beings such as agency, awareness and reflexivity (requiring transparency on how data are collected and distributed) (principles 1 and 4);
C. Create and maintain trust and confidentiality between users and providers (all 4 principles);
D. Embed inclusiveness in design (principle 3);
E. Facilitate public health actions and user engagement related to IoT for health (principles 1, 3 and 4).

Nevertheless, in an LSP Project, apart from the overarching ethical principles guidelines (see also Supplementary Note 1), each pilot should refer to their respective ethical committee and follow their own ethical procedure. As such, the Ethical approval procedure is an integral part of the research process as it aims to protect both researchers and participants. Participants should be provided with enough details to make informed, autonomous decisions^17^. Therefore, while respecting shared principles, within GATEKEEPER each pilot has followed the procedures requested by their local Ethical Committees for submitting their ethical approvals, preparing the required documentation following the official forms and the languages locally requested.

During the preparation of the pilot application, a multiformity of ethical application procedures and documents to be submitted to different Local Ethical Review Boards (LERBs) different for each pilot and sometimes for each RUC emerged, resulting in a jeopardised situation, which revealed a complexity of the procedural mechanism that it seemed appropriate to rethink starting from a unified perspective^18^. In fact, it was noted that even if the framework of existing principles is very well-structured and satisfying, there is a lack of tools that can concretely, and in a harmonized way, guide the application of such principles in the management of a LSP research project.

For all the reasons anticipated, this manuscript provides an overview of the ethical management strategy implemented in the GATEKEEPER project and the proposed idea of building a common European ethical legal framework that could serve as a model for supporting and guiding the management of LSP research projects in the future.

## Results

### The Unified Ethics Application Form

Due to its nature, GATEKEEPER, one of the biggest and most highly financed EU research projects, could not refer to other similar experiences for ethical legal management and the development of an architecture of strategies in a manner that would lead to consistent results and, therefore, had to design its own framework.

In order to better comprehend the results and ensure alignment with the methodology structure described below, the results were divided into two main phases, as follows:

The Phase 1 involved:

A. A mapping was performed of all the legal and regulatory referrals for the GATEKEEPER Project (see Supplementary Table 2), along with a list of the ethical principles that would be applicable to the project’s activities which were disseminated to the project’s partners (Supplementary Table 1) with the aim of building the ethical-legal GATEKEEPER framework.
B. In order to facilitate the performance of ethical risk assessments on pilot sites, a checklist was created to better guide the partners in the procedure to be followed and the points to be taken into consideration when performing their own ethical impact assessments (Supplementary Table 4).
C. Based on the literature review conducted, the lack of specific scientific literature on the topic of the ethical management of LSP research projects became apparent, which, in turn, led to the project’s attempts and mitigation actions described in this section, which were progressively adjusted to ensure compliance with ethical requirements.
D. - E) - F) The absence of adequate guidelines on the management and governance of LSP led to the pilots being requested to fill in the Questionnaire on the ethical procedure and to collect the Ethical Applications and the English Summaries. Those documents highlighted two crucial elements within the management of LSP research projects: (1) the incredibly complex situation among different pilot sites which not only have different ethical forms and procedures to be followed, but even different LERBs (i.e., of the Hospital, of the University, Regional ones, National ones etc.) which jeopardises the adequate performance of their activities, and (2) the plurality of languages of the documents collected, which implies that the management team must be comprised of members with multilingual competences in order to revise in-depth the documentation.

As demonstrated above, the ethical procedures in the various sites differed significantly depending, among others, on the type of institution performing it and the location. Given this lack of homogeneity even though the information requested and provided was similar, there were two main consequences on a project coordination level, notably that (a) the project’s coordination team could not provide unified pilot-wide support in preparing the required information and documentation, and proceeding without delays, and (b) further monitoring and validation of the ethics procedures at a project level were hindered.

As a result, the methodological analysis led to the identification of the need for a unique application form for research ethics that can serve as a model for the universalisation of ethical procedures in research projects, particularly relevant for LSP ones (Phase 2). The main objective behind the unique application form for research ethics is to overcome the challenges posed by the co-existence of multiple languages and formats required to complete the Ethical Approval forms and procedures. As such, the form has been designed to be used by pilots in the course of individual organisation procedures towards the Ethical Approvals, and replace existing local solutions, that are specific to one organisation exclusively. Such a step, albeit ambitious, would require a single adjustment of the organisation’s framework that would facilitate all relevant future actions and its participation in research projects where ethical elements would need to be both duly considered and reported in a verifiable manner.

This form establishes an ethical legal framework in which all the pilot sites can be represented, without compromising the need for ad hoc adjustments in accordance with national legislation and other regulatory requirements. The above-described comparative analysis served as the baseline in order to identify the elements that needed to be included in the Unified Ethics Application Form.

The form is complemented by supporting documentation that may be requested, which can always be expanded according to the needs of each organisation. Overall, the form is divided into four main sections, as follows:

A. The first section lays down the foundation of general information with regard to the research project requesting Ethical Approval;
B. The second section provides a detailed description of the research, including information on its duration, participation criteria, and funding;
C. The third section focuses on the main ethical and legal considerations to be taken into consideration, as well as on the research project’s management from an ethical and legal perspective;
D. The final section includes additional provisions that may be applicable according to national dispositions and ad hoc requirements of each research organisation.

Each element included in the Unified Ethics Approval Form is accompanied by further guidance on which information should be particularly considered and how to fill in the requested information. This additional guidance is meant to provide further clarity surrounded the information required, facilitate researchers filling in the form and ensuring no information is omitted.

**Fig. 1:**
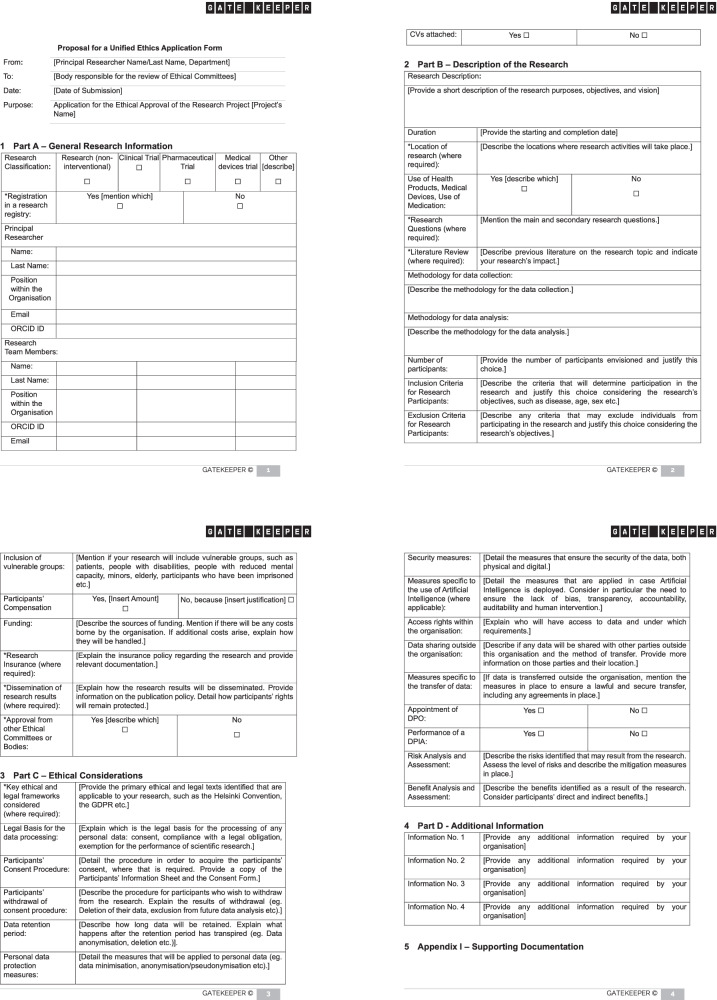
Novel Ethical Approval Form. Overview of the proposed model for an Ethical Approval Form.

The form’s overview can be found below (Fig. 1), while the complete form is submitted as Supplementary Table 5.

Taking the above into consideration, the form furthermore serves as a tool for the streamlining, simplification, and acceleration of ethical procedures in research projects, both at the beginning of the projects and during potential revisions. At the same time, it constitutes a reference framework for the ethical principles and legal requirements to be respected in EU research projects related to the use of technologies for human health, in alignment with the principles enshrined in European and International regulations, declarations, and guidelines.

## Discussion

The GATEKEEPER project provided the possibility to reflect on the ethical and legal aspects of an LSP research project on two distinct levels of analysis: the first concerning the ethical *procedures* for a research project, and, the second examining, at a macro level, how to *manage* LSP projects.

As anticipated, one of the main considerations that emerged during the management of the ethical aspects present in GATEKEEPER was the fact that, despite the existence of a number of evident gaps that could negatively impact the success of the project, there were no formal guidelines or references to support their management. As such, the main gaps underlined were:

1. The lack of universal tools to manage an LSP project from an ethical and legal perspective. On the contrary, the existence of a number of guidelines and different procedures per country or per pilot implies the need for every research project to have Legal and Ethical Managers to identify, map, and interpret them for each research project. Even in the latter scenario, it is necessary to have clear and unambiguous guidelines when performing their duties, without having to repeatedly establish them on the basis of general Declarations of Principles or non-binding documents originating from a number of different bodies.
2. The extreme differences in the ethical application procedures per each pilot and even RUCs, and of the type of LERBs the pilots should address. This is problematic because it multiplies the documents, while also requiring the repetition of information, thus increasing the amount of work due from the researcher and slowing down the process of obtainment of the approvals. This is considered a crucial problem by the research community, which tried to argue in favour of a common perspective^18^. Yet, the establishment of a uniform ethical and legal approach that can serve as a model for the Ethical Approval procedures was never explicitly addressed.
3. The fact that per each pilot site or RUC, the ethical approval in the local language was sufficient and its translation was not required in any official document, not even when it was made known that it would be shared with the LSP project management or the European Commission. This consideration does not have a blind critique nor suspicion that the pilot sites involved could have hidden behind the language’s unambiguousness to conceal a lack of adherence to ethical principles or lack of compliance with ethics and law. On the contrary, it is well known that all the pilots’ sites for their ethical application follow the instructions of LERBs, which in order to be recognised as such, must follow international commonly agreed ethical and legal requirements. The committees’ official status guarantees the trustworthiness of their work. Nevertheless, it is true that LSP Projects may also include non-European countries or countries not adhering to values and principles shared in the EU territory. For this reason, adapting the Ethical Approval Forms and relevant documentation in an international language, such as English is a priority. This does not have to be necessarily on a pilot level, but it is paramount to forward to the management of these projects and the Commission, not only the summary, as was the case in the GATEKEEPER project, but of all the documents’ content^19^.
4. The response time of the Ethics Committees or respective Bodies is not predictable and varies according to the workload of their members, as well as the number of meetings planned. Any delays in response times, which can exceed a year, can be exacerbated by the committee’s request for amendments or clarifications^20^. This has a significant impact on research projects, especially if they are multicentric or large-scale because a delay in the pilot phase ensues that could hinder the research project’s success^21^. This falls outside the scope of responsibility of the consortium of researchers, often required to deal more with soliciting the LERBs rather than carrying out their tasks. Therefore, the proposal for a single framework for all pilots of European research projects could represent a streamlining of the procedure and a speeding up of practices which will positively impact the future of the research^22^.
5. The COVID-19 pandemic outbreak, as anticipated, further slowed down the work of the LERBs, which prioritised the evaluation of the research projects related to the pandemic, postponing all the other ethical applications. This halt continued for almost two years (2020–2021) and negatively affected the deployment and development of the GATEKEEPER project. However, this not only confirms that the multiformity of LERBs complicates and slows down an already complex process but also highlights that a uniform approach would have expedited the evaluation contemporarily the projects related to COVID-19 and the others without a long delay, in case this hiatus is to occur again in the future. It is anticipated that the COVID-19 pandemic was only one of the first global health emergencies and, for this reason, scientists, policymakers, and regulators are working to be “prepared” for future pandemics^23^. This stresses the importance of not repeating the errors of the past and finding new strategies to accelerate the evaluation of research projects in times of emergency. The idea of a unique legal and ethical framework could be considered part of this process of preparing for the future of research projects, in particular related to health.

Moreover, there were striking differences noted regarding the pilots’ Ethical Approval Forms themselves. For reference, based on the comparative analysis performed, it became evident that the approaches widely differ based on jurisdiction and the field of activities of each pilot. In particular, the following was noted:

A. Where the pilot was performed in the context of a University Hospital, the requirements tended to be stricter and more extensive compared to pilots performed outside the context of university hospitals and clinics. This is understandable considering not only the frequency in which university hospitals and clinics are subject to such procedures but also the overall sensitive nature of their activities. Considering that their performance of research activities almost always involves patients, vulnerable populations, health data, clinical and medical studies, or a combination of those, it is logical that a higher level of protection and attention to detail is imperative.
B. Pilots performed outside of the EU, albeit still in the EEA, were inclined to impose stricter requirements and request additional information/documentation, frequently simulating the information requested and the structure of the Horizon 2020 and Horizon Europe programmes. The proposed Ethical Application Form has taken into consideration those requirements, even though they do not reflect the situation in the entirety of the pilots, so as to ensure that all pilot sites are adequately represented. As such, the proposed Ethical Application Form leaves room for the application and compliance with national dispositions that may be applicable.
C. The description and analysis of measures and safeguards adopted throughout the research with regard to privacy and personal data protection have been a central focal point in the procedures of all pilots. Given the transition to a data-centred economy and the ever-increasing processing of personal data, ensuring data subjects’ privacy is becoming more and more essential to democratic societies. This focus on privacy and personal data protection reflects precisely the need to balance innovation and human rights, without either of them excluding the other.
D. Most Ethical Review Boards required specific and explicit information on funding sources, aiming at ensuring the impartiality of research. By validating that funding is secured through objective and impartial sources, and that adequate measures are in place, it is ensured that the results are unbiased and trustworthy.
E. In most pilots, a long list of supporting documents was requested, further exaggerating the bureaucratic element of the procedure. Such documents most frequently included the detailed research protocol, the patient information leaflet, and the consent forms, as well as documentation regarding funding and insurance. In many of the pilots, the researchers’ curriculum vitae was also requested, along with a formal declaration of assumption of responsibility with regard to the project.

Despite the above, there remained a minimum of information that was required in the majority of the pilots. In particular, those common elements that were requested regarding the research were:

1. The research classification as a research or clinical trial;
2. The introduction of the research team;
3. The description of the research, its objectives and expected outcomes;
4. The research’s duration;
5. The methodology for data collection and analysis;
6. The description of the intended participants’ characteristics;
7. The number of participants.

Taking the above into consideration, our proposal for a Unified Ethics Application Form has focused on three main elements: (i) compliance with all of the pilots’ procedures, (ii) simplification, (iii) an easy-to-fill application format that thoroughly guides the researcher as to the information they have to provide in order to proceed to the submission of their research project for an Ethical Approval. By creating three primary sections that differentiate the information between general, research description and ethical considerations, and additional ad hoc information, there is a two-fold benefit for the organisation; on one hand, the competent Committee can more easily mark which of the additional information it deems necessary to examine, and, on the other, the researcher is in a better position to intuitively fill in the required information, avoiding repetitions.

Finally, by establishing a common form along pilot sites, as proposed above, the procedures for validating the Ethical Approvals within the research project are also facilitated. Thanks to their simplicity and unified approach, the documents can be easily translated and shared with the project’s management and legal team to serve as the baseline for the design of further compliance activities, including the performance of a Data Protection Impact Assessment (DPIA), risk and conformity assessments, as required by relevant legislation in each case. As a result and considering the Ethical Approval’s importance for the demonstration of compliance, the project’s activities towards regulatory and ethical compliance become more easily auditable, traceable, and transparent.

As already discussed, the GATEKEEPER LSP is a federation of multicentre longitudinal cohort studies, aiming at demonstrating the effectiveness and the cost-effectiveness of Key Enabling Technologies (KETs) such as AI, big-data, and Internet of Things (IoT) for the prevention of adverse events and the management of health in later life. Therefore, while respecting shared principles, each pilot was obliged to follow the procedures requested by their LERBs for submitting their ethical approvals and preparing the required documentation, in alignment with the official forms and in the languages locally requested, leading to a lack of homogeneity and an increase in complexity. This situation demonstrated the fundamental importance of Ethical and Legal management in support of each pilot and the need for a unified strategy in this kind of project (LSP), which can speed up the process of the ethical application and approval, ensuring adherence to commonly recognised regulatory and ethical references.

This paper not only presents the process of managing the ethical and regulatory aspects of an LSP project that is a rarity in the international research landscape and can be considered a reference model for future similar research projects on technology on human health but it also proposes a unified ethical and legal framework that can be considered an exemplary tool to standardise procedures, overcome the existing situation, and speed up the procedural hurdles that could jeopardise the success of a research project, particularly on a large scale.

The lack of existing information on LSP projects and in particular on the management of ethical issues, as well as the need to overcome local particularisms to tend to a unique perspective among the partners of a research project, make this work unique and worthy of further studies.

## Methods

### Top down and bottom-up approaches

**Fig. 2:**
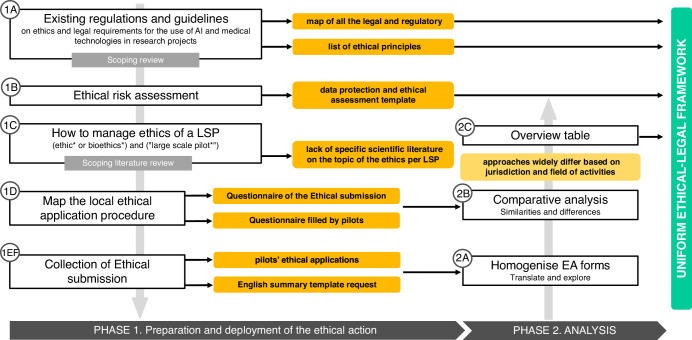
Methodology workflow. Workflow for establishing the unified ethical-legal framework.

In order to provide a clear picture of all the tools assessed for the deployment of the ethical and legal management of the GATEKEEPER project, the process was divided into two main phases as described in Fig. 2.

**Phase 1**: Preparation and deployment of the ethical action – top-down approach:

A. A Scoping review of existing regulations and guidelines was performed on the internet and specific websites (e.g., EU, WHO) on the theme of ethics and legal requirements for the use of AI and medical technologies in research projects^6^.
B. During the early stages of the project, an Ethical Risk Assessment was performed (and prevised some Mitigation Strategies). As stated previously, one of the aims of GATEKEEPER is to be able to contribute to its field of innovation and research by producing and ethical impact assessment model that could be deployed to evaluate e-health processes and products. In order to do so, a data protection and ethical assessment template was co-created and filled with the pilot representatives.
C. A Scoping Literature Review was performed on PubMed and it aimed at gathering knowledge relative to the management of the ethics of LSP projects. The search string, i.e., “(ethic* OR bioethics*) AND (“large scale pilot*”)” returned only 4 publications, out of which 2 were excluded because out of topic^24^. Considering the scarce number of papers extracted from the systematic approach, a scoping search on the web was also performed and a few more papers and documents on the topic were added (Supplementary Table 3).
D. With the aim of mapping the local ethical application procedure of each pilot, specific actions were performed (with the continuous support of the pilots’ sites to ethical and legal expert staff). In particular a Questionnaire on the ethical procedure steps required by each Ethical Committee per different pilot site (Supplementary Note 2) was created and handed out to the pilot representatives.
E. After obtaining information on the procedure to be followed per each pilot site, the collection of Ethical submissions started. In that stage, each pilot representative was supported in the writing and preparation of the document submitted to their reference ethical committee and for possible amendments, when needed.
F. Given the difference within the documents related to the ethical submission in terms of templates and languages (each pilot sent to the GK Management the original version of the submission in their own local language), it was decided to request that all pilot representatives fill in the WHO model of “Recommended format for a ‘research protocol’” (https://www.who.int/groups/research-ethics-review-committee/recommended-format-for-a-research-protocol/) in the English language, which was named English Summary.

**Phase 2:** Analysis at the late stages of the project and proposal of the unique framework – bottom-up approach.

Taking the above into consideration, and in order to reach the proposal for a common Ethical Approval framework for health-related research activities, described below, a bottom-up approach was adopted. Benefiting from the procedures followed by partners within the GATEKEEPER project to secure the respective Ethical Approval and be able to perform their research tasks, a set of similarities and differences was identified. Said observations served as the baseline for a common Ethical Approval framework that could significantly facilitate the relevant procedures, without compromising adequate examination of each case ad-hoc.

In particular, in order to achieve the above-described objectives, the following procedure was performed:

A. The first step required to be able to compare the relevant procedures followed by each pilot for their Ethical Approval was the translation of all Ethical Approval application forms from their original language into English, since each Ethical Review Board performs all related activities in the official language of the region where it is based.
B. After their translation, a comparative analysis was performed in-depth, aiming at extracting any similarities and/or discrepancies in the approaches followed in each pilot’s case. The extensive analysis extracted, compiled, and compared all information provided across the pilot sites for their respective Ethical Approvals.
C. In order to ensure that the observations could be safely monitored and validated, while leaving room for reflection, an extensive comparative table was created (Supplementary Table 6). Said table provided a complete overview of all aspects regarding the Ethical Approval procedure, as was described in each application form, ranging from the form’s required format to the necessary supporting documentation. The table demonstrates all the unique Ethical Approval Forms that were requested in the context of the Pilots and has excluded the Ethical Approval Forms that were submitted in the same body using the same form for different RUCs. A shorter version of the comparative table (Table 2) can be found below, where the requirements are marked with a mark (✓), while the mark including the plus symbol (✓ + ) indicates the requirement that researchers provide more extensive information on the topic.

**Table 2:** Comparative table of Ethical Approval Form required information

Pilot	Aragon	Basque Country	Cyprus	Greece			Poland	Puglia			Saxony	UK	
Partners	1	1	1	1	2	3	1	1	2	3	1	1	2
Directed to	✓	✓	✓	✓	✓	✓	✓	✓	✓	✓	✓	✓	✓
Research Participants	✓	✓	✓	✓	✓ +	✓	✓ +	✓	✓	✓	✓	✓	✓ +
Literature Review	✓	-	-	-	-	-	-	-	-	-	-	✓	-
Research classification (Clinical trial, Research, Study etc)	✓	✓	✓	✓	✓	✓	✓	✓	✓	✓	✓	✓	✓ +
Research description, duration and participants’ characteristics for recruitment	✓	✓	✓	✓	-	✓	✓	✓ +	✓	✓	✓ +	✓ +	✓ +
Number of research participants mentioned	✓	✓	✓	-	-	✓	✓	-	-	-	-	-	✓
Use of Health products/ Medical devices/ Medical Intervention	✓	✓	-	-	-	-	✓	✓ +	-	-	✓	✓	✓
Methodology of data collection and analysis description	✓	✓	✓	-	-	-	✓	✓	✓	✓	✓	✓	✓
Personal data and protection measures	✓ +	✓	✓	✓	✓	✓	✓	✓	✓	✓ +	✓ +	✓ +	✓ +
Consent procedure	✓	-	✓	-	-	-	✓	✓	✓	✓	-	✓	✓
Risk Analysis and Assessment	✓	-	✓	-	-	-	✓	-	-	-	✓	✓	✓
Benefits Assessment	✓	-	✓	-	-	-	✓	-		✓	✓	✓	✓
Funding Sources	✓	✓	✓	✓	-	✓	-	✓ +	✓	✓	✓	✓	✓

Where an element was requested in at least six ethical approval forms, this was translated in a generally applicable requirement and information that would be required.

Nonetheless, and in order to achieve the representation of all pilot sites, the remaining requirements that were required in five or less ethical approval forms, were handled in the following manner, namely they were:

1. Clustered, where possible based on the level of similarity, in one requirement (eg. Information on the inclusion of minors and on the inclusion of imprisoned individuals were taken into consideration in the requirement to report the inclusion of vulnerable groups); or
2. Reported in a simple yes/no manner, requiring that these elements are merely reported, not imposing any obligation to that end, unless otherwise required by the organisation’s internal procedures (eg. Information on whether a DPO has been appointed or whether a DPIA has been performed); or
3. Marked with an asterisk, meaning that the requirement may only be applicable under certain circumstances and in certain jurisdictions.

The above-described process underlined the need for further actions to propose a harmonised approach to the procedures followed by the pilots for the demonstration of their compliance with the applicable ethical and legal requirements, which is required in order to obtain the Ethical Approval and proceed with their research activities.

### Reporting summary

Further information on research design is available in the Nature Research Reporting Summary linked to this article.

### Supplementary information

Supplementary Material

Reporting Summary

## Data Availability

The datasets used and/or analysed during the current study available from the corresponding author on reasonable request.
