# Supplementary material for: Towards a common European ethical and legal framework for conducting clinical research: the GATEKEEPER experience
Source: NPJ Digit Med. 2024 Apr 13;7:95. doi: 10.1038/s41746-024-01092-x (PMC11016076; doi:10.1038/s41746-024-01092-x)
Supplement: Supplementary file 1 — Supplementary Material [file 41746_2024_1092_MOESM1_ESM.pdf]

Supplementary Note 1

The GATEKEEPER Project envisaged a series of activities for the management of legal and ethical aspect, as described in the Description of Action (DoA). The relevant process is described as follows:

1. Preparation and Assessment of a Legal, Ethics and Privacy Protection (LEPP) Management manual, where guiding principles and the procedures on privacy, data protection, legal issues, and ethical challenges were defined. This document also provided an annual evaluation of risks, including an ethical risks compilation (based on a template distributed to all partners), followed by suggested mitigation measures.

2. Collection of all the ethical approval from each pilot and each RUC.

3. The building of a Policy, Legal and Gender Board which, among its other duties, focused on ensuring that the GATEKEEPER project pilots are executed in an ethically sound manner and in compliance with relevant national and international ethical requirements for trials involving patients. This board was formed by legal experts from industry and demand users of the consortium (one per local pilot), as well as by policymakers and gender equality officers from the demand cites in the consortium.

4. The identification of an Ethical, Legal and Gender Issues Manager (ELGM), who also served as the Data Manager, responsible (with the support of the Ethical, Legal and Gender Board) for the management of the following issues: a) Legal aspects: the legal issues associated to the deployment of GATEKEEPER tools and actions (e.g. IPR, data protection and access, privacy issues, ethical aspects, etc.), b) Policy issues: how new policies could help innovative smart living technologies get users acceptance and market uptake, Gender issues: the ELGM will be responsible to supervise the implementation the gender equality policy of the project and c) ethical, security and data management concerns in data management.

Apart from that, there was continuous interaction among pilots and LSP management through online and in-person meetings during the plenaries aimed at underlining and dealing with the existing gaps.

Supplementary Note 2

Any study involving human participants requires an ethical review assessment and to apply for ethical approval, especially when personal data are collected and processed. For this reason, scholars are required to refer to their local (i.e., Research Institute, Hospital, Local Trust, and/or Regional) Ethics Committees following procedures requiring specific application forms and local guidance. These Ethic Committees are required to comply with European and International

guidelines and in this regard is possible to find an online manual for the Ethics Appraisal Procedure[1].

The following pages draw information from the responses each pilot gave to the following questionnaire.

QUESTIONNAIRE PROVIDED TO PILOTS:

I - Please, describe the Ethical Approval (EA) Procedure by answering these questions:

1. Do you need to submit an EA?	yes no
1.a If not, why? And/or what is your alternative strategy?	
1.b If yes, do you already have an EA form?	yes no
1.b.1 If yes, what type of EA do you have?	
1.b.2 If not, what kind of EA do you need?	
2. Explain the EA procedure followed in order to obtain ethical approval (meeting, definition of the protocols...)	
3.Who do you have to submit your documents to [REC (Research Ethics Committee), HRA (Health Research Authority), Hospital Ethical Committee (EC), Regional EC, other]?	
4.Obtaining of EA (possible changes due to suggestions of EC).	

II- **Documents for the Ethical Approval:**

Attach the following documents, if applicable:	a) main document (summary of the clinical study, study design, methodology & data analysis, recruitment…)<br>b) participant information leaflet<br>c) informed consent<br>d) ethical approval

III- **Ethical Approval**

Please attach your Ethical Approval	

IV- **Others**

Please specify if you have provided temporary documents (if these have been submitted to the reference body), what are the reasons and when you will send the final document.	

[1] https://ec.europa.eu/research/participants/docs/h2020-funding-guide/cross-cutting-issues/ethics_en.htm

Supplementary Table 1

Ethical principles informing the Gatekeeper project

Principles	Application
**Respect for Persons**<br>It includes respect for autonomy and personal integrity. Respect for persons demands that subjects enter into the research voluntarily and with adequate information	• Inform and seek advice if there are concern related to the integrity and quality of the project and pilots<br>• Treat patients as autonomous agents and respect their right to determine their own best interest.<br>• Participation in the pilots is voluntary and follows from informed consent.<br>• Enable participants to make reasoned informed choices and decisions<br>• The collection of informed consent must follow three steps:<br>-*Information*: detailed information of the pilot, including potential benefits, risks and limitations, must be provided;<br>-*Comprehension*: The information must be given both verbally and written in clear language, in a precise and calm manner and in the proper context. Participants should be invited to ask any questions they may have;<br>-*Voluntariness*: The informed consent form must stress that participation is voluntary and that participants are free to withdraw at any time at their own discretion and at no cost (without reprehension)
**Beneficence**<br>Persons are treated in ethical manner not only by respecting their decisions and protecting them from harm, but also by making efforts to secure their well-being	• Explain the limitations of the Gatekeeper pilots, particularly in terms of probably personal and/or health benefits during and after the pilot;<br>• Inform of possible clinical incidental findings prior of

	informed consent form and how these will be handled;<br>• Follow standard clinical practices for consulting relevant specialist and for informing patients of clinical incidental findings and take appropriate actions;<br>• Maximise probable benefits and minimise possible harms;<br>• Continuously assess probable risk and benefits. The probable benefits must be deemed higher than the probable harm;<br>• Put the health and welfare of participants at the highest priority
**Justice**<br>It included the process of selecting participants in a justifiable manner	• The selection of participants must be fair and equal i.e. inclusion/exclusion in the trial must not be denied a person without good reason but must be based on reason directly related to the objectives of the pilot
**Respect for confidentiality and privacy**<br>Legal compliance should be guaranteed, but also a moral and ethical commitment to respect confidentiality and privacy	• The Gatekeeper project (all project partners) must treat participant information with confidentiality;<br>• Participants may exercise control over personal information by consenting to, or withholding consent for, the collection, use and/or disclosure, modification, loss or theft;<br>• Implement the Privacy by design principle;<br>• Adherence to national and international regulations on privacy and data protection

**Transparency**<br>Open and transparent information shall be guaranteed to researchers and patients	• The Gatekeeper project must be as transparent as possible in explaining its goals;<br>• Access to public documents of the project shall be guaranteed;
**Sustainability**<br>The solutions proposed by the project are sustainable in the medium and long term	• The Gatekeeper project must strive to find sustainable solutions both for the hospitals and for the patients;<br>• The Gatekeeper project must aim to minimize the impact of the suggested solutions on the environment and on the use of energy

Supplementary Table 2

Relevant legislations and soft laws

Below is a list of relevant normative sources that Gatekeeper will taking into account in its various phases and to finalize the compliance checks and the ethical impact assessment. Any arising ethical issue will be handled taking into account the principles of the following legal instruments:

-Helsinki Declaration of 1964 (revised version 2004);

-European Convention of Human Rights;

-Rules of the Convention of the Council of Europe for the protection of individuals (automatic processing of personal data);

-EU General Data Protection Regulation;

-Charter of fundamental rights of the European Union;

Ethical and Social issue	Field	Law/Directive

**Human Dignity and Integrity of user**	Human Rights	-Universal Declaration of Human Rights (United Nations);<br>-Convention for the Protection of Human Rights and Fundamental Freedoms (Council of Europe);<br>-European Charter for Fundamental Rights (European Union);<br>-Draft recommendation of the Council of Europe on the promotion of the human rights of older persons;<br>-European Charter of the Rights of Older People in need of long-term care and assistance;
**Privacy**	Data Protection	-Regulation (EU) 2016/679 of the European Parliament and of the Council of 27 April 2016 on the protection of natural persons with regard to the processing of personal data and on the free movement of such data, and repealing Directive 95/46/EC (General Data Protection Regulation);<br>-Directive (EU) 2016/680 of the European Parliament and of the Council of 27 April 2016 on the protection of natural persons with regard to the processing of personal data by competent authorities for the purposes of the prevention, investigation, detection or prosecution of criminal offences or the execution of criminal penalties, and on the free movement of such data, and

		repealing Council Framework Decision 2008/977/JHA;<br>-Directive 2006/24/EC of the European Parliament and of the Council of 15 March 2006 on the retention of data generated or processed in connection with the provision of publicly available electronic communication services or of public communications networks and amending Directive 2002/58/EC;<br>-Directive 2002/58/EC of the European Parliament and of the Council concerning the processing of personal data and the protection of privacy in the electronic communications sector;<br>-Regulation (EU) 2017/745 of the European Parliament and of the Council of 5 April 2017 on medical devices, amending Directive 2001/83/EC, Regulation (EC) 178/2002 and Regulation (EC) 1223/2009 and repealing Council Directives 90/385/EEC and 93/42/EEC;<br>-Regulation (EU) 2017/746 of the European Parliament and of the Council of 5 April 2017 on in vitro diagnostic medical devices and repealing Directive 98/79/EC and Commission Decision 2010/227/EU
**Bioethics and clinical trials**	Medical Research	-World Medical Association Declaration of

		Helsinki-Ethical Principles for Medical Research involving human subjects;<br>-Opinion on the processing of health data by Article 29 Data Protection Working Party;<br>-Universal Declaration on Bioethics and Human Rights;<br>-Directive 2001/20/EC on the approximation of the laws, regulations and administrative provisions of the Member States relating to the implementation of good clinical practice in the conduct of clinical trials on medicinal products for human use;<br>-Convention for the Protection of Human Rights and Dignity of the Human Being with regard to the Application of biology and Medicine: Convention on Human Rights and Biomedicine (and Guide for Research Ethics Committee Members);<br>-Charter for the Rights of Older People in Clinical Trials;<br>-Council Directive 90/385/EEC of 20 June 1990 on the approximation of the laws of the Member States relating to Active Implantable Medical Devices as amended by Directive 2007/47/EC of 5 September 2007;<br>-Council Directive 93/42/EEC of 14 June 1993

		concerning Medical Devices as amended by Directive 2007/47/EC of 5 September 2007;<br>-Directive 98/79/EC on In Vitro Diagnostic Medical Devices as amended by Directive 2007/47/EC of 5 September 2007;<br>-Commission Regulation (EU) 207/2012 of 9 March 2012 on electronic instructions for use of medical devices;<br>-Directive 2001/83/EC of the European Parliament and of the Council of 6 November 2001 on the Community code relating to medicinal products of human use
**Disability**	Accessibility	-Disability Rights Commission: Guidelines for ethical research (2004);<br>-UN Convention on the Rights of persons with disabilities;<br>-Accessibility Act
**New Technologies**	Liability and Safety	-Directive 85/374/EC on liability for defective products as amended by Directive 1999/34/EC;<br>-Directive 2011/24/EU on the application of patients' rights in cross-border healthcare;<br>-Directive 90/385/EEC on active implantable medical devices and Directive 93/42/EEC on medical devices and Directive 98/79/EC on in vitro diagnostic medical devices;<br>-RoHS Directive 2002/95/EC of the European Parliament and of

		the Council of 27 January 2003 on the restriction of the use of certain hazardous substances in electrical and electronic equipment;<br>-Directive 98/34/EC of the European Parliament and of the Council of 20 July 1998 amended by Directive 98/34/EC laying down a procedure for the provision of information in the field of technical standards and regulation and of rules on information society services
**Covid19**	Epidemic	-European Commission recommendation on a common Union toolbox for the use of technology and data to combat and exit from the COVID-19 crisis, in particular concerning mobile applications and the use of anonymised mobility data, 8/4/2020;<br>-E-health network Mobile applications to support contact tracing in the EU's fight against COVID-19. Common EU Toolbox for Member States, 15/04/2020;<br><br>-Communication from the Commission, Guidance on Apps supporting the fight against COVID 19 pandemic in relation to data protection, 16/4/2020;<br><br>-European Commission's Joint European Roadmap Towards Lifting COVID-19 Containment Measures;

		-European Data Protection Board, Statement on the processing of personal data in the context of COVID-19 outbreak, 19/3/2020;<br>-European Data Protection Board, Guidelines 3/2020 on the processing of personal data concerning health for the purpose of scientific research in the context of the COVID-19 outbreak, 21/4/2020;<br><br>-European Data Protection Board, Guidelines 4/2020 on the use of location data and contact tracing tools in the context of the COVID19 outbreak;

Table 2: Relevant normative sources

Supplementary Table 3

The following table show the results of the scoping review on the Ethics of Large Scale pilot projects.

**Author**	**Title**	**Journal info**
Nalin M, Baroni I, Faiella G, Romano M, Matrisciano F, Gelenbe E, Martinez DM, Dumortier J, Natsiavas P, Votis K, Koutkias V,	The European cross-border health data exchange roadmap: Case study in the Italian setting.	J Biomed Inform. 2019 Jun;94:103183. doi: 10.1016/j.jbi.2019.103183. Epub 2019 Apr 19. PMID: 31009760.

Tzovaras D, Clemente F.		
Segkouli S, Fico G, Vera-Muñoz C, Lecumberri M, Voulgaridis A, Triantafyllidis A, Sala P, Nunziata S, Campanini N, Montanari E, Morton S, Duclos A, Cocchi F, Nava MD, de Lorenzo T, Chalkia E, Loukea M, Colomer JBM, Dafoulas GE, Guillén S, Arredondo Waldmeyer MT, Votis K.	Ethical Decision Making in Iot Data Driven Research: A Case Study of a Large-Scale Pilot.	Healthcare (Basel). 2022 May 23;10(5):957. doi: 10.3390/healthcare10050957. PMID: 35628094; PMCID: PMC9141539.
Lehana Thabane, Jinhui Ma, Rong Chu, Ji Cheng, Afisi Ismaila, Lorena P Rios, Reid Robson, Marroon Thabane, Lora Giangregorio & Charles H Goldsmith	A tutorial on pilot studies: the what, why and how	BMC medical research methodology 10 (2010): 1-10.
Drajic, D., Andersson, K., Zhang, K., Stembert, N., Malmberg, K., Brékine, A., ... & Waeben, J	User engagement for large scale pilots in the Internet of Things	2019 14th International Conference on Advanced Technologies, Systems and Services in Telecommunications (TELSIKS). IEEE, 2019. p. 46-53.
Dantas, C., Machado, N., Ortet, S., Leandro, F., Burnard, M.,	The Iterative Model of Ethical Analysis for Large-Scale Implementation Of ICT Solutions.	Translational Medicine@ UniSa, 2020, 23: 1.

Grünloh, C., ... & Pocs, M		
Verbeke, K., Krawczyk, T., Baeyens, D., Piasecki, J., & Borry, P.	Informed Consent and Debriefing When Deceiving Participants: A Systematic Review of Research Ethics Guidelines	Journal of Empirical Research on Human Research Ethics, 2023, 15562646231173477.
The Council for International Organizations of Medical Sciences (CIOMS)	International ethical guidelines for health-related research involving humans	2016 - https://cioms.ch/publications/product/international-ethical-guidelines-for-health-related-research-involving-humans/
EU Commission	Horizon 2020 Online Manual: Ethics	https://ec.europa.eu/research/participants/docs/h2020-funding-guide/cross-cutting-issues/ethics_en.htm
WHO	Standards and operational guidance for ethics review of health-related research with human participants	https://www.who.int/publications/i/item/9789241502948

Supplementary Table 4

Check list	Compliance (yes/no/not applicable)	Comments
**Informed consent** – Participants have been given detailed information about the pilot, including its purpose, limitations and potential benefits and risks		
**Informed consent**-The information has been given verbally with supporting written information and in clear language		

**Informed consent**-Participants have had the opportunity to rethink if they want to participate or not		
**Informed consent**-The decided informed consent procedure has been followed and copies of all participants signed informed consent forms have been forwarded to the responsible person		
**Autonomy**-The voluntariness of the participation has been stressed as have the option to withdraw at any time without any repercussion		
**Autonomy**-The pilot will not use a technology/device that constrains a person or curtails their freedom of movement or association		
**Autonomy-**Participants are able to control the technologies/devices used for monitoring, i.e. they can switch them off or choose not to send data		
**Autonomy-**In the recruitment process and during the pilot participants are/have been treated as autonomous agents and their right to determine their own best interest is respected		
**Dignity-** Participants' personal information is treated with confidentiality		
**Dignity-**The pilot recognize and respect the right of participants to lead a life of dignity and independence and to participate in social and cultural life		
**Stigmatisation-**Upmost efforts have been made to ensure that the least intrusive, physically and aesthetically, devices and technologies are used in the trial		
**Stigmatisation-**The pilot do not require participants to use a technology that marks them in some way as cognitively or physically disabled		

**Inclusion-**Participants' needs and requirements have been defined and used to guide the selection of technologies/devices as usability is a priority		
**Transparency-**Researchers have been transparent explaining the project goals		
**Transparency-**Full access to the public documents of the project is guaranteed		
**Sustainability-**An initial sustainability check has been performed		

Preliminary ethical impact assessment for the Gatekeeper platform in its design phase

Questions

	**Guiding question**	**Substantive Conceptual question**
Q 1	Does this platform threaten the freedom of individual humans?	Does this platform alter an individual's freedom of movement?<br>Does this platform interfere intentionally with the formation of expression or beliefs?
Q 2	Does this platform threaten the natural equality of persons?	Are expected benefits divided between groups for reasons not associated with difference in use?
Q 3	Does this platform restrict the exercise of a dignified human life?	Will this platform reduce the chance of life choices (e.g. nudges) of individuals in ways they are not fully aware of?
Q 4	Does this platform seek to change the way in which individuals' reason?	Will this platform restrict access to information?<br>Will this platform promote specific decision-making schemes the users will not be aware of?
Q 5	Does this platform alter the exercise of human moral conscience?	Will this platform promote specific visions of a good life?
Q 6	Is this platform explicitly designed to create or exacerbate inequalities between individuals or groups?	Are expected benefits divided between groups for reasons nor associated with differences in use?
Q 7	Is this platform intended to create tiers of persons on the basis of social, International, or political factors?	Does this limit the rights of any individuals or groups based upon race?<br>Does this limit the rights of any individuals or groups based upon biological sex?

Q 8	Does this platform restrict the enjoyment of basic human rights?	Does this limit the natural life of an individual? Is this designed to enhance or augment the natural life of an individual?<br>Does this restrict an individual's opportunity to exercise liberties?

	**Guiding question –Answer**	**Substantive Conceptual question-Answer**
A 1	No	No, No
A 2	No	No
A 3	No	No
A 4	Maybe, some services can promote healthy habits	No, No
A 5	No	Maybe, some services can promote healthy habits
A 6	No	No
A 7	No	No, No
A 8	No	No, No

Supplementary Table 5:

Proposal for a Unified Ethics Application Form

From:	[Principal Researcher Name/Last Name, Department]
To:	[Body responsible for the review of Ethical Committees]
Date:	[Date of Submission]
Purpose:	Application for the Ethical Approval of the Research Project [Project's Name]

1 Part A – General Research Information

Research Classification:	Research (non-interventional)	Clinical Trial ☐	Pharmaceutical Trial	Medical devices trial	Other [describe]

	☐		☐	☐	☐
*Registration in a research registry:	Yes [mention which] ☐		No ☐		
Principal Researcher					
Name:					
Last Name:					
Position within the Organisation					
Email					
ORCID ID					
Research Team Members:					

Name:			
Last Name:			
Position within the Organisation			
ORCID ID			
Email			
CVs attached:	Yes ☐	No ☐	

2 Part B – Description of the Research

Research Description**:**	
[Provide a short description of the research purposes, objectives, and vision]	
Duration	[Provide the starting and completion date]
*Location of research (where required):	[Describe the locations where research activities will take place.]

Use of Health Products, Medical Devices, Use of Medication:	Yes [describe which] ☐	No ☐
*Research Questions (where required):	[Mention the main and secondary research questions.]	
*Literature Review (where required):	[Describe previous literature on the research topic and indicate your research's impact.]	
Methodology for data collection:		
[Describe the methodology for the data collection.]		
Methodology for data analysis:		
[Describe the methodology for the data analysis.]		
Number of participants:	[Provide the number of participants envisioned and justify this choice.]	
Inclusion Criteria for Research Participants:	[Describe the criteria that will determine participation in the research and justify this choice considering the research's objectives, such as disease, age, sex etc.]	
Exclusion Criteria for Research Participants:	[Describe any criteria that may exclude individuals from participating in the research and justify this choice considering the research's objectives.]	
Inclusion of vulnerable groups:	[Mention if your research will include vulnerable groups, such as patients, people with disabilities, people with reduced mental capacity, minors, elderly, participants who have been imprisoned etc.]	
Participants' Compensation	Yes, [Insert Amount] ☐	No, because [insert justification] ☐
Funding:	[Describe the sources of funding. Mention if there will be any costs borne by the organisation. If additional costs arise, explain how they will be handled.]	
*Research Insurance (where required):	[Explain the insurance policy regarding the research and provide relevant documentation.]	
*Dissemination of research results (where required):	[Explain how the research results will be disseminated. Provide information on the publication policy. Detail how participants' rights will remain protected.]	

*Approval from other Ethical Committees or Bodies:	Yes [describe which] ☐	No ☐

3 Part C – Ethical Considerations

*Key ethical and legal frameworks considered (where required):	[Provide the primary ethical and legal texts identified that are applicable to your research, such as the Helsinki Convention, the GDPR etc.]
Legal Basis for the data processing:	[Explain which is the legal basis for the processing of any personal data: consent, compliance with a legal obligation, exemption for the performance of scientific research.]
Participants' Consent Procedure:	[Detail the procedure in order to acquire the participants' consent, where that is required. Provide a copy of the Participants' Information Sheet and the Consent Form.]
Participants' withdrawal of consent procedure:	[Describe the procedure for participants who wish to withdraw from the research. Explain the results of withdrawal (eg. Deletion of their data, exclusion from future data analysis etc).]
Data retention period:	[Describe how long data will be retained. Explain what happens after the retention period has transpired (eg. Data anonymisation, deletion etc.)].
Personal data protection measures:	[Detail the measures that will be applied to personal data (eg. data minimisation, anonymisation/pseudonymisation etc).]
Security measures:	[Detail the measures that ensure the security of the data, both physical and digital.]
Measures specific to the use of Artificial Intelligence (where applicable):	[Detail the measures that are applied in case Artificial Intelligence is deployed. Consider in particular the need to ensure the lack of bias, transparency, accountability, auditability and human intervention.]
Access rights within the organisation:	[Explain who will have access to data and under which requirements.]
Data sharing outside the organisation:	[Describe if any data will be shared with other parties outside this organisation and the method of transfer. Provide more information on those parties and their location.]
Measures specific to the transfer of data:	[If data is transferred outside the organisation, mention the measures in place to ensure a lawful and secure transfer, including any agreements in place.]

Appointment of DPO:	Yes ☐	No ☐
Performance of a DPIA:	Yes ☐	No ☐
Risk Analysis and Assessment:	[Describe the risks identified that may result from the research. Assess the level of risks and describe the mitigation measures in place.]	
Benefit Analysis and Assessment:	[Describe the benefits identified as a result of the research. Consider participants' direct and indirect benefits.]	

4 Part D - Additional Information

Information No. 1	[Provide any additional information required by your organisation]
Information No. 2	[Provide any additional information required by your organisation]
Information No. 3	[Provide any additional information required by your organisation]
Information No. 4	[Provide any additional information required by your organisation]

5 Appendix I – Supporting Documentation

Supplementary table 6

Pilot	Aragon	Basque Country	Cyprus	Greece			Poland	Puglia			Saxony	UK	
Partners	1	1	1	1	2	3	1	1	2	3	1	1	2
Format	Application	Application	Application	Letter	Application	Application	Application	Application	Application	Application	Application	Application	Application
Directed to	✓	✓	✓	✓	✓	✓	✓	✓	✓	✓	✓	✓	✓
Research Participants	✓	✓	✓	✓	✓ +	✓	✓ +	✓	✓	✓	✓	✓	✓ +
Researchers' CV	✓	✓	✓	-	-	-	-	-	-	-	-	-	-

Literature Review	✓	-	-	-	-	-	-	-	-	-	-	✓	-
Research classification (Clinical trial, Research, Study etc)	✓	✓	✓	✓	✓	✓	✓	✓	✓	✓	✓	✓	✓ +
Research description	✓	✓	✓	✓	-	✓	✓	✓ +	✓	✓	✓ +	✓ +	✓ +
Duration	✓	✓	✓	✓	-	✓	✓	✓	✓	✓	✓	✓	✓
Number of research participants mentioned	✓	✓	✓	-	-	✓	✓	-	-	-	-	-	✓
Use of Health products/ Medical devices/ Medical Intervention	✓	✓	-	-	-	-	✓	✓ +	-	-	✓	✓	✓
Methodology of data collection and analysis description	✓	✓	✓	-	-	-	✓	✓	✓	✓	✓	✓	✓

Patients’ characteristics for recruitment	✓	✓	✓	✓	-	✓	✓	✓ +	✓	✓	✓	✓	✓ +
Legal framework mentioned	-	✓	✓	✓	-	-	-	-	-	-	✓	✓	-
Personal data and protection measures	✓ +	✓	✓	✓	✓	✓	✓	✓	✓	✓ +	✓ +	✓ +	✓ +
Consent procedure	✓	-	✓	-	-	-	✓	✓	✓	✓	-	✓	✓
DPO Appointed	-	-	-	✓	-	-	-	-	-	-	✓	-	-
Inclusion of minors	✓	-	✓	-	-	-	-	-	-	-	-	✓	✓
Inclusion of participants who have been imprisoned	-	-	-	-	-	-	-	-	-	-	-	-	✓
Withdrawal of consent	-	-	-	-	-	-	✓	✓	-	-	-	-	✓
Patients’ complaint procedure	-	-	✓	-	-	-	-	-	-	-	-	-	-
DPIA Conducted	-	-	-	✓	-	-	-	-	-	-	-	✓	-

Risk Analysis and Assessment	✓	-	✓	-	-	-	✓	-	-	-	✓	✓	✓
Benefits Assessment	✓	-	✓	-	-	-	✓	-		✓	✓	✓	✓
Funding Sources	✓	✓	✓	✓	-	✓	-	✓ +	✓	✓	✓	✓	✓
Additional Costs	-	-	✓	✓	-	✓	-			✓	-	-	✓
Research Insurance	-	-	-	-	-	-	-	-	-	-	✓	-	✓
Publication of research results	-	-	-	-	-	-	-	✓	-	-	-	-	-

Supporting Documentation	1.Commitment of the principal investigator and collaborators<br>2.CV of all researchers<br>3.Information Sheet for the Participant and Informed Consent (See template on the CEICA website) or HIP/CI waiver request and authorization for access to records (for example, medical records).<br>4.Financial report/ source of financing<br>5.Authorization for the use of resources (Annex II)<br>6.Certificate of insurance policy (if applicable).<br>7.CE marking certificate and technical sheet (if it is a medical device).<br>8.Pseudonymous data ONLY if it complies with section 3.3 (use of secondary source data)	1.Patient information leaflet<br>2.Informed consent form<br>3.Research protocol<br>4.Principal investigator commitment<br>5.Conformity of the services	1.Research protocol<br>2.Consent forms	1.Research Protocol<br>2.Case Report Form, and:<br>a.ADL Scale<br>b.IADL scale<br>c.White card for reporting Medical technologies products incidents for users		1.Research protocol<br>2.Information document without the need for consent<br>3. Consent form<br>4.Funding information<br>5.Department/Lab Permit<br>6.Information of the research project	1. Study protocol,<br>2. Information for the examined / patient<br>2a. Information for the test / patient regarding genetic testing<br>3. Form of informed consent of the examined / patient to participate in the study<br>3a. Form of informed consent of the examined / patient to perform genetic tests<br>4. List of centers participating in the study.<br>5. A photocopy of the liability insurance<br>6. Statement of the examined / patient on the acceptance of the terms of third-party liability insurance<br>7. Statement of the examined / patient expressing consent to the processing of personal data<br>8. Principal Investigator's CV (signed).<br>9. Consent of the	1. Request to authorise the study<br>2. Assumption of responsibility and research direction<br>3. Declaration regarding the existence of a conflict of interest<br>4. Research Summary<br>5. Financial Agreement Proposal<br>6. Assumption of Responsibility<br>7. Benefit of the research Declaration<br>8. Declaration regarding the observational nature of the study	1. Letter of intent signed by the Director of the department where the study will take place (see attached CE evaluation request, to be customized according to the study)<br>2. Study Protocol (including date and version)<br>3. Study Synopsis in Italian (including date and version)<br>4. Informed Consent Form and Patient Information Sheet (including date and version). The consents form is customarily checked in advance, for the data protection aspect, by the privacy office<br>5.Decla	1. General Information<br>1.1. Letter of Request for study evaluation<br>1.2. Annex 1 Summary Sheet of Experimentation characteristics (if applicable)<br>1.3. Annex 2 Declaration of responsibility and suitability for the study<br>1.4. Annex 3 Cost Declaration<br>1.5. Annex 4 Application for clinical experimentation authorization<br>2.1. Study Information and Consent Form<br>3.1. Study Protocol in Italian<br>3.2. Synopsis in Italian	1. Study protocol,<br>2. Informed consent forms,<br>3. Opnion on insurance coverage,<br>4. Declaration of assumption of costs or fee reduction/ exemption	1.Consent form<br>2.Project Outline<br>3.DPIA<br>4.Project registration and Risk Checklist	1. Declaration by Chief Investigator,<br>2. Declaration by the sponsor's representative

	(available on the CEICA website)<br><br>9.Responsible declaration in the case of observational studies with drugs (EOM) without commercial interest, if applicable (see model on the CEICA website).<br><br>10.If the study is carried out at the University, authorization from the University must be presented for the processing of personal data, unless data from the Aragonese Health System						director of the hospital (centre) to conduct the examination or of a person authorized by the director.<br>10. Other (specify the characteristics of the submitted documents).		ration of Responsibility and Suitability for the study (Attachment 2)<br><br>6. Cost Declaration (Attachment 3)<br><br>7.Declaration of absence of conflicts of interest (Attachment 5)<br><br>8. Case Report Form (CRF) (if available)<br><br>9. Principal Investigator's Curriculum Vitae (updated and signed)	3.3. Case Report Form (CRF) - List of information to be collected<br>4.1. Annex 5 Researcher's Declaration of Public Conflict of Interest<br>4.2. CV of the Principal Investigator			
